# Osteoblast Viability of Liquid Smoke Rice Hull and Nanoparticles Form as Periodontitis Treatment

**DOI:** 10.1055/s-0042-1745772

**Published:** 2022-07-08

**Authors:** Ira Arundina, Indeswati Diyatri, Wisnu S. Juliastuti, Theresia I. Budhy, Meircurius D. C. Surboyo, Benni Iskandar, Sheryn M. Ramaniasari, Azzahra S. A. Moelyanto, Gustiadi Saputra

**Affiliations:** 1 Department of Oral Biology, Faculty of Dental Medicine, Universitas Airlangga, Surabaya, Indonesia; 2 Department of Oral Pathology and Maxillofacial, Faculty of Dental Medicine, Universitas Airlangga, Surabaya, Indonesia; 3 Department of Oral Medicine, Faculty of Dental Medicine, Universitas Airlangga, Surabaya, Indonesia; 4 School of Pharmacy, College of Pharmacy, Taipei Medical University, Taipei, Taiwan; 5 Sekolah Tinggi Ilmu Farmasi, Pekanbaru, Riau, Indonesia; 6 Bachelor Dental Science Program, Faculty of Dental Medicine, Universitas Airlangga, Surabaya, Indonesia; 7 Magister of Immunology, Faculty of Medicine, Universitas Airlangga, Surabaya, Indonesia

**Keywords:** nanoparticles, liquid smoke, rice husk, osteoblast and human and health

## Abstract

**Objective** Rice husk liquid smoke nanoparticles have the potential to be developed as a drug because they have anti-inflammatory effects that can modulate the process of osteoblast stimulation through osteoblast stimulation by thorough small size and enter cells easily. The osteoblast is the key of alveolar regeneration in periodontitis treatment. This present study analyzed the differences of liquid smoke rice husk and nanoparticles of liquid smoke rice husk on osteoblast viability as periodontitis treatment

**Materials and Methods** The liquid smoke rice husk was obtained from the pyrolysis process. The nanoparticles were made with chitosan, maltodextrin, and difference of concentration of liquid smoke rice husk (such as 1, 2.5, 5, 7.5, 10, 12.5, 15, and 17.5%). The viability of osteoblast was analyzed by 2,5-diphenyl-2H-tetrazolium bromide (MTT) assay.

**Statistical Analysis** The data were analyzed using independent t-test to analyze the differences between liquid smoke rice husk and nanoparticles of liquid smoke rice husk, the significant was set a *p* <0.05.

**Result** The nanoparticles of liquid smoke rice husk showed higher osteoblast viability compared liquid smoke rice husk. The nanoparticles' concentration of 5, 7.5, and 10% showed higher osteoblast viability compared liquid smoke rice husk ( *p* = 0.002, 0.000, and 0.001, respectively).

**Conclusion** The nanoparticles of liquid smoke rice husk showed higher viability of osteoblast. This confirmed that the nanoparticles were able to reduce the toxicity in the higher concentration of liquid smoke of rice husk.

## Introduction

Rice husks can be processed into liquid smoke that be used in the field of health. 1 Liquid smoke is a compound that results from pyrolysis 2 which contains organic components, such as phenols and acetic acid, that possessed antioxidant and antimicrobial properties. 3 Topical administration of liquid smoke rice husk inhibits the secretion of proinflammatory cytokines, 4 induce the growth factors secretion that play a role during the ulcer healing, and periodontitis. 1 The active compounds in the liquid smoke of rice husks can work optimally if they reach the therapeutic target effectively and efficiently. This can be achieved using nanotechnology, namely, nanoparticles.

Nanoparticles are materials that are approximately 1 to 100 nm in size. 5 In the biomedical field, nanoparticles are used as carriers of drugs, imaging, as well as therapies, and diagnostic tools. 6 Nanoparticles are used as drug carriers by dissolving, trapping, encapsulating, absorbing, or chemically attaching active ingredients. 7 Nanoparticles can increase drug absorption, thereby increasing its bioavailability. 8 Nanoparticles can improve stability and the ability to protect labile substances against degradation factors. 9 Nanoparticles can circulate in the bloodstream, across tissues, enter cells, 5 increases permeability when passing through biological barriers, such as blood-brain barriers, and intestinal barriers, so that a compound can effectively reach therapeutic targets. 6

Nanoparticles of liquid smoke rice husk have the potential to be developed as a drug because they have anti-inflammatory effects that can modulate the process of osteogenesis through the inhibition of proinflammatory cytokines and their small size, so that they can enter cells easily. 10 The absorbance and bioviability of high nanoparticles has a possible risk of unwanted nanoparticle accumulation in the human body, so it is important to know their toxic potential. 8 MTT assay is a test that often used to assess toxicity *in vitro* , 11 and to measure cellular metabolic activity as an indicator of cell viability, proliferation, and cytotoxicity. 12

The liquid smoke of rice husk has been shown a lower toxicity 13 and inhibit the periodontitis bacteria. 14 15 The liquid form has disadvantage to penetrate into periodontal tissue due to its solubilities and release properties. This present study examined the toxicity of liquid smoke of rice husk and nanoparticles of liquid smoke rice husk as periodontitis in the osteoblast cell culture.

## Materials and Methods

### Chemicals and Reagent

MTT (3-[4,5-dimethylthiazol]-2.5 diphenylterazolium bromide assay; Thiazole Blue Tetrazolium, M2128, Sigma Aldrich, Missouri, United States); phosphate buffer saline (PBS; Bioenno Tech, California, United States); dimethyl sulfoxide (DMSO; AnalaR, BDH limited, Poole, England); sterile water (API IPHA, IPHA laboratory, Bandung-Indonesia); Roswell Park Memorial Institute (RPMI) 1,640 (Invitrogen Life Technologies Inc., Burlington, ON, Canada); 10% (v/v) fetal bovine serum (FBS; Gibco, Carlsbad, California, United States); chitosan (Bio-chitosan, Indonesia); and maltodextrine (Qinghuadong Lihua Starch, China).

### Liquid Smoke of Rice Husk

The liquid smoke of rice husk used in this study was obtained with pyrolysis process from 1,760 g of rice hull which was air dried at room temperature following a previous study. 16 Liquid smoke of rice husk was diluted by sterile water for make concentration of 1, 2.5, 5, 7.5, 10, 12.5, 15, and 17.5%.

### Nanoparticles of Liquid Smoke Rice Husks

Each concentration of liquid smoke of rice husk (1, 2.5, 5, 7.5, 10, 12.5, 15, and 17.5%) was made as nanoparticles with chitosan and maltodextrin. Chitosan (1.5% w/v) and maltodextrin (8.5% w/v) are dispersed in a solution of glacial acetic acid water (1.0% v/v). The chitosan–maltodextrin nanoparticles are made by complexation of chitosan polyelectrolyte with maltodextrin and additional chitosan ionic glass with sodium tripolyphosphate (TPP) anion. Chitosan and maltodextrin are dissolved in liquid smoke rice husks. Sodium TPP (1.0 mg/mL) is added to the mixture and stirred using a magnetic stirrer at 200 rpm for 30 minutes at room temperature. The nanoparticles are isolated by centrifugation at a speed of 3,000 rpm in a 50 mL cone tube for 30 minutes at room temperature. Supernatants is discarded and nanoparticles filtered in a vacuum using Whatman no. 2. The nanoparticle solution is heated at 50°C into a water bath for 15 minutes and homogenized using a speed rotor–stator homogenizer at 5,200 rpm for 2.5 minutes. 17

### Osteoblast Cell Culture

7F2 cells maintained in Dulbecco's Modified Eagle Medium (DMEM) is supplemented with 10% Fetal Bovine Serum, Penicillin G (100 μg/mL), and Streptomycin (100 μg/mL). Cells were plated onto 75 cm ^2^ culture flasks and allowed to grow to confluence. Cultures were maintained at 37°C in a humidified atmosphere of 5% CO _2_ in air with culture media changed every 48 to 72 hours. Detachment of cells was accomplished by the addition of a trypsin–ethylenediaminetetraacetic acid (EDTA) solution in phosphate-buffered saline (PBS). 18

### Treatment of Osteoblast Cell Cultures

7F2 cells that have been distributed in wells are divided into 12 groups, namely, group 1 as positive control using cell control containing cells in culture is considered a 100% of cells, group 2 as negative control using media control containing culture media alone is considered a percentage of cells 0%, groups 3 to 12 are exposed to liquid smoke nanoparticles of rice husks with a concentration of 0.5, 1, 2.5, 5, 7.5, 10, 12.5, 15, 17. 5, and 100%. The microplate is then incredated for 24 hours at 37°C, then removed from the incubator. 15

### Osteoblast Viability

Osteoblast viability was performed with MTT assay which is a colorimetric test to assess cell metabolic activity. Osteoblast cells (6 × 10 ^3^ cells/wells) was added into a 96-well microplate. After osteoblast cells were cultured for 24 hours, then were treated with LS-RH 1, 2.5, 5, 7.5, 10, 12.5, 15, 17.5, and 100% at 20 μL. Then incubation was performed for 24 hours. 10-μL MTT (0.5 mg/mL) was added into each well and incubated at 37°C for 4 hours. Next, the medium from each well was discarded, then 100 mL DMSO was added to dissolve formazan salt and each well would be read with a microplate reader (Bio-Rad, model 550) at 490 nm. Each test was replicated three times independently. The viability of osteoblast was calculated using formula:

Viability (%) = (sample OD − blank OD) / (control OD – blank OD) × 100%

Information:
Proliferation: value of optical density (OD) for each sample.
Sample OD: value of OD after each test.
Media: value of OD on the average of each media control.
Cell: value of OD on average of cell control.

### Statistical Analysis

The data were shown in the form of mean ± standard deviation (X ± SD) for each group and each measurement. Then, the data were analyzed using independent *t* -test to analyze the differences between liquid smoke rice husk and nanoparticles of liquid smoke rice husk the significance was set at *p* < 0.05.

## Result

### Characteristics of Nanoparticles of Liquid Smoke Rice Husks

The characterization of nanoparticles of liquid smoke rice husk was describe as average size and Polydispersity (PDi) index as mentioned in Table 1 .

**Table 1:** Characteristics of liquid smoke rice husk nanoparticles

Sample	*Z* -average (d **ays** .nm)	Pdi
1	33.03	0.543
2	31.55	0.626
3	33.94	0.566
4	33.00	0.557
5	32.69	0.633
Average	32.84 ± 0.86	0.585 ± 0.415

The liquid smoke that was obtained from rice husk nanoparticles has a bright yellow color. The acidity of the liquid smoke from rice hulls was 3.41 and the density was 1.04 g/mL. The size of average was 32.84 ± 0.86 days.nm with PdI was 0.585 ± 0.415 ( Table 1 ).

### Osteoblast Viability of Liquid Smoke Rice Husk

**Fig. 1:**
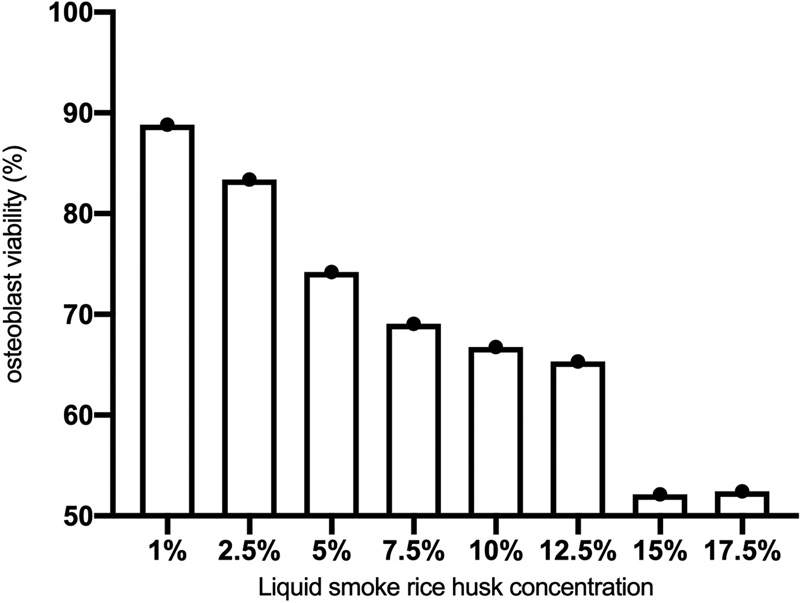
The viability of osteoblast in liquid smoke rice husk.

The osteoblast viability with liquid smoke of rice husk was presented on Fig. 1 . Liquid smoke of rice husk concentration of 1, 2.5, 5, 7.5, 10, and 12.5% showed an osteoblast viability more than 60%. The highest viability was found in the liquid smoke of rice husk concentration of 1% (88.83%). In other hand, liquid smoke of rice husk concentration of 15 and 17.5% showed the lowest osteoblast viability (52.15 and 52.44%).

### Osteoblast Viability of Nanoparticles of Liquid Smoke Rice Husk

**Fig. 2:**
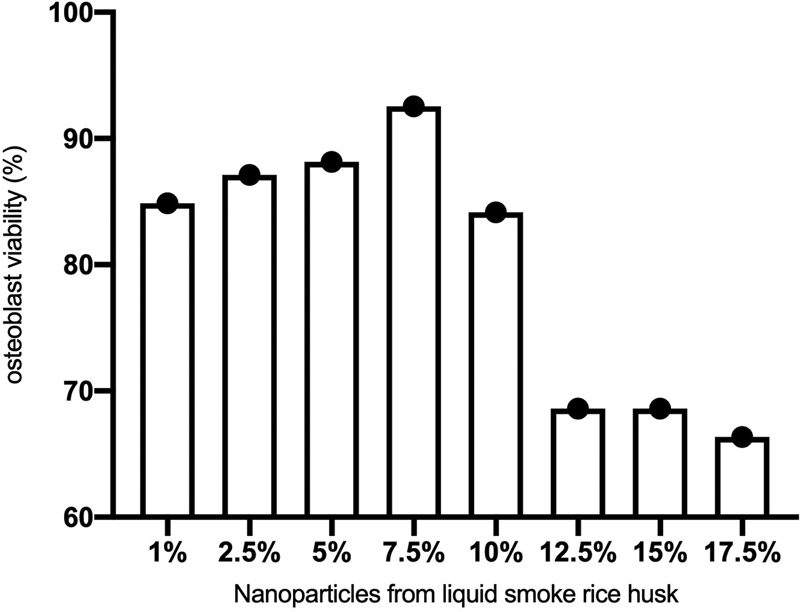
The viability of osteoblast in nanoparticles of liquid smoke of rice husk.

The osteoblast viability with nanoparticles of liquid smoke of rice husk was presented on Fig. 2 . Liquid smoke of rice husk concentration of 1, 2.5, 5, 7.5, 10, 12.5, 15, and 17.5% showed an osteoblast viability more than 60%. The highest viability was found in the liquid smoke of rice husk concentration of 7.5% (92.83%).

### The Differences of Osteoblast Viability of Liquid Smoke Rice Husk and Nanoparticles of Liquid Smoke Rice Husk

The differences of osteoblast viability between liquid smoke rice husk and nanoparticles of liquid smoke rice husk were observed in the concentration of 5, 7.5, 10, 15, and 17.5%. The nanoparticles of liquid smoke rice husk concentration of 5, 7.5, and 10% showed higher osteoblast viability compared liquid smoke rice husk ( *p* = 0.002, 0.000, and 0.001). The nanoparticles of liquid smoke rice husk concentration of 15 and 17.5% also showed higher osteoblast viability compared liquid smoke rice husk ( *p* = 0.002).

## Discussion

Rice ( *Oryza sativa* L.) is a major food source in most South Asia countries, especially Indonesia. One of the by-product of this plant is rice hull that can be utilized to be a liquid smoke through distillation process. 19 This liquid smoke contains many phenolic compounds which has potent antioxidant properties that can bind to free radicals. 20 The toxicity property of this liquid smoke was assessed on the osteoblast's viability by the MTT assay. The MTT assay is based on the reduction of the yellow tetrazolium salt to purple formazan crystals by dehydrogenase enzymes secreted from the mitochondria of metabolically active cells. The amount of purple formazan crystals formed is proportional to the number of viable cells. 21 The result of MTT assay stated that liquid smoke rice husk has high percentage of osteoblast viability, this indicate that this liquid did not have toxic effect. However, the percentage of osteoblast viability decrease with the higher liquid smoke rice husk concentration, but not for nanoparticles of liquid smoke rice husk increase osteoblast viability from concentration of 1 to 7.5%.

The active compound of liquid smoke rice husk might originate from guaiacol and its derivate. 20 Guaiacol (2-methoxyphenol) is a phenolic compound with two functional groups, hydroxyl (–OH) group and methoxy (–OCH3) group. 22 Guaiacol have the ability to scavenge free radicals through its high ionization potential. Guaiacol also can give hydrogen atoms (H ^+^ ) to –OH to form H _2_ O and inhibit generation of superoxide radicals (O _2_ –). 23 Another guaiacol derivate that already identified inside liquid smoke rice husk is 4-ethyl-2-methoxyphenol (EMP). 24 EMP has stronger antioxidant properties than guaiacol and a potent NO scavenger and provides the anti-inflammatory properties. 25 The anti-inflammatory of liquid smoke related to its ability to inhibit nuclear factor (NF)-kB, 20 its proinflammatory cytokines, and upregulates the Nuclear Respiratory Factor 1 (NRF1). 26 By this properties, it might be the reason the osteoblast viability remain more than 50%.

The manufacture of liquid smoke nanoparticles of rice husks through encapsulation with chitosan biopolymer material will form a cross-bond with the TPP. TPP acts as a cross-linking agent resulting in nanoparticles of a smaller and more stable size. 27 Chitosan can increase the flexibility of molecular structure so that it will improve some properties such as solubility or mucoadhesion. 28 Chitosan is a positively charged molecule that interacts with the negatively charged cell membrane (∼70 mV) due to the ion exchange between intracellular and extracellular media mediated by the Na ^+^ /K+ pump. Because positively charged nanoparticles that use chitosan material more easily enter the cell. 29 The combination of liquid smoke rice husk with chitosan and maltodextrin produces a good nanoparticles with the average size was 32.84 ± 0.86 days.nm with PdI was 0.585 ± 0.415. This characteristic also provides higher osteoblast viability compared with liquid smoke rice husk.

The active compounds in nanoparticles liquid smoke rice husk can provide optimally picked effects because nanoparticles can circulate in the bloodstream, across tissues, and enter cells, 5 and also increase permeability when passing through biological barriers such as blood–brain barriers and intestinal barriers, so that a compound can effectively reach therapeutic targets. 6 Another advantage of nanoparticles is the increased affinity of the system due to an increase in the contact surface area at the same amount. 30 The nanoparticles of liquid smoke rice husk increased osteoblast viability may induced the secretion from growth factors, such as fibroblast growth factors, that can affect the proliferation of osteoblast cells. 1 FGF activates a large number of signaling pathways, such as phospholipase γ (PLC γ), and extracellular receptor kinase (ERK), and phosphatidylinositol-3-kinase (PI3K) that will activate RUNX2 and cause increased expression of osteoblastogenic markers such as Alkaline Phosphatase (ALP), Osteocalcine (OCN), Collagen type 1 (COL-1). All osteoblastogenic marker expressions play a role in osteoblast proliferation and osteoblasts gene expression, especially in periodontitis. 31

The higher osteoblast viability provides an evidence that the untoxicity, both liquid smoke rice husk and nanoparticles, of liquid smoke rice husk as alternative and natural medicine can be an opportunity in dentistry

## Conclusion

The nanoparticles of liquid smoke rice husk showed higher viability of osteoblast. This confirmed that the nanoparticles are able to reduce the toxicity in the higher concentration of liquid smoke of rice husk. Current in vitro study provided evidence that liquid smoke of rice husk has possibility to use as periodontitis treatment.
